# The Role of Intestinal Permeability in Gastrointestinal Disorders and Current Methods of Evaluation

**DOI:** 10.3389/fnut.2021.717925

**Published:** 2021-08-26

**Authors:** Tim Vanuytsel, Jan Tack, Ricard Farre

**Affiliations:** ^1^Department of Chronic Diseases, Translational Research Center for Gastrointestinal Disorders, Metabolism and Ageing, Catholic University Leuven, Leuven, Belgium; ^2^Division of Gastroenterology and Hepatology, Leuven University Hospital, Leuven, Belgium

**Keywords:** intestinal permeability, GI diseases, coeliac disease, chronic liver disease, inflammatory bowel disease, bile acid malabsorption, paracellular route, transcellular route

## Abstract

An increased intestinal permeability has been described in various gastrointestinal and non-gastrointestinal disorders. Nevertheless, the concept and definition of intestinal permeability is relatively broad and includes not only an altered paracellular route, regulated by tight junction proteins, but also the transcellular route involving membrane transporters and channels, and endocytic mechanisms. Paracellular intestinal permeability can be assessed *in vivo* by using different molecules (e.g., sugars, polyethylene glycols, ^51^Cr-EDTA) and *ex vivo* in Ussing chambers combining electrophysiology and probes of different molecular sizes. The latter is still the gold standard technique for assessing the epithelial barrier function, whereas *in vivo* techniques, including putative blood biomarkers such as intestinal fatty acid-binding protein and zonulin, are broadly used despite limitations. In the second part of the review, the current evidence of the role of impaired barrier function in the pathophysiology of selected gastrointestinal and liver diseases is discussed. Celiac disease is one of the conditions with the best evidence for impaired barrier function playing a crucial role with zonulin as its proposed regulator. Increased permeability is clearly present in inflammatory bowel disease, but the question of whether this is a primary event or a consequence of inflammation remains unsolved. The gut-liver axis with a crucial role in impaired intestinal barrier function is increasingly recognized in chronic alcoholic and metabolic liver disease. Finally, the current evidence does not support an important role for increased permeability in bile acid diarrhea.

## Introduction

The concept of the “leaky gut” also known as increased intestinal permeability has been described many years ago and is currently receiving increasing attention in the scientific literature but also in the media because of its proposed associations with numerous conditions that do not necessarily affect the gastrointestinal (GI) tract, such as asthma, Alzheimer's disease, diabetes among others. This is the reason why different interventions such as probiotics, and dietary modifications intended to ameliorate the leaky gut, are suggested as potential treatments.

The principle behind the “leaky gut” theory is that endogenous (e.g., psychological stress, intestinal inflammation) and exogenous factors (e.g., diet, alcohol intake) can increase intestinal permeability, allowing the entrance of food antigens, commensal or pathogenic bacteria and bacterial components into the lamina propria and later on into the systemic circulation, provoking the systemic inflammation described in different disease conditions. The vast majority of studies evaluate the intestinal permeability of the paracellular route, despite the presence of other routes of transepithelial transport that are potentially more relevant in gastrointestinal disorders.

To better comprehend the role of an increased intestinal permeability in GI diseases, it is crucial to understand some basic concepts on how different molecules cross the intestinal epithelium and how intestinal permeability can be evaluated in humans.

## Routes of Transport in the Epithelium: Paracellular, Transcellular, Transporter Mediated and Endocytosis

**Figure 1:**
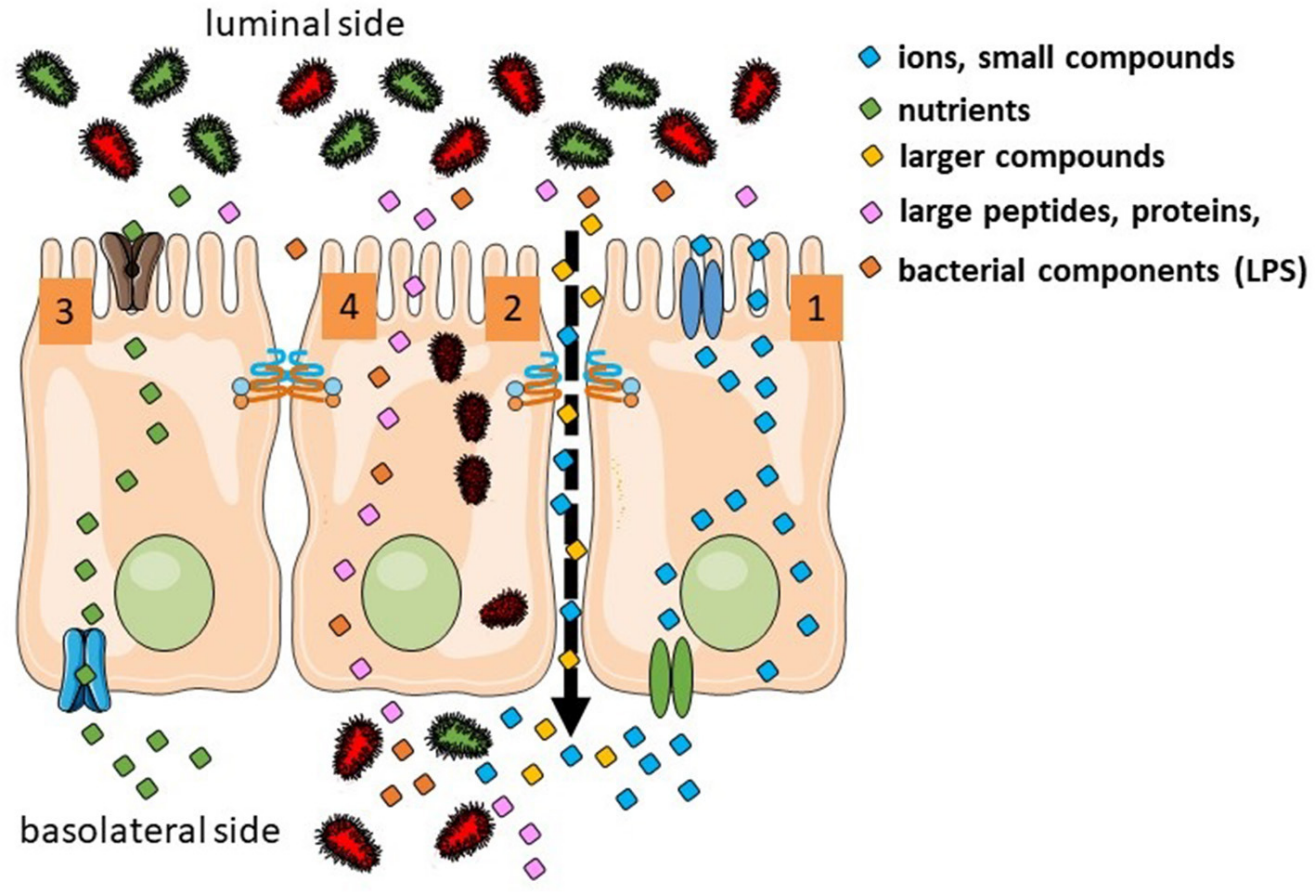
Transport routes in the intestinal epithelium. Transcellular transport of ions is controlled by transporters in the apical and basolateral surfaces (1). Ion, water, and larger hydrophilic compounds use the paracellular pathway (2). Sugars, amino acids, and vitamins use active transport (3). Large molecules and whole bacteria are endocytosed into vesicles (4).

There are several routes for luminal products to cross the intestinal epithelium. They depend of the size, hydrophobicity and other physico-chemical properties (Figure 1). Overall there are four different pathways: (a) the transcellular route used by small hydrophilic and lipophilic compounds; (b) the paracellular route used by ions, water and larger hydrophilic compounds from ~400–600 Da up to 10–20 kDa and that cannot cross transcellularly [regulated by the tight junction (TJ) proteins]; (c) transcellular active transport used by nutrients as sugars, amino acids and vitamins that require specific transporters and energy and (d) endocytosis and basolateral exocytosis via vesicles are used by larger peptides and proteins, large bacterial components or even whole bacteria (1, 2). From a physiological point of view, the paracellular route and the transcellular endocytic route are the two relevant pathways in the context of the leaky gut. Nevertheless, most of the *in vivo* studies assessing human intestinal permeability (oral administration of sugars or other probes later detected in blood or urine) mainly target the paracellular pathway.

The transcellular endocytic pathways is then understudied but has been shown very relevant in several diseases GI and non-GI disorders. A very illustrative example is the increased bacterial translocation (BT) observed in numerous pathologies and that is defined as the migration of viable micro-organisms and bacterial components from the gut lumen toward the mesenteric lymph nodes (MLNs) and extra-intestinal sites such as the peritoneal cavity, liver, spleen and kidney (3) among others. Importantly, in some pathological conditions as intestinal ischemia and advanced liver disease, BT can have fatal consequences. Lipopolysaccharide (LPS) is by far the most studied bacterial protein, located on the surface of Gram-negative bacteria (size of 10–20 kDa). LPS has been found in higher concentrations in the systemic circulation of patients with cirrhosis (4) and other diseases as inflammatory bowel disease (IBD) (5) and even in patients with diarrhea-predominant irritable bowel syndrome (IBS-D) (6).

### The Paracellular Route

Evidences for the role of TJs as key regulators in the epithelial barrier are based historically on observation from two very different fields: electron microscopy (EM) and electrophysiology. In the most apical region of the epithelium there is an intercellular gap (around 90 Å) named *zonula occludens* or tight junctions, followed by the *zonula adherens* (gap of 200 Å), followed by the *macula adherens* or desmosomes (gap of ~240 Å) (7). More than 50 year ago, Hans Ussing initially described by performing electrophysiology in the frog skin that the TJ structure is a dynamic and permeant barrier that in physiological conditions is also permeable to ions and plays a role in processes like water absorption and secretion (8). Moreover, early experiments in rat kidney tubules using transmission EM showed that the flux of large molecules across the paracellular route is selectively regulated by complex tight junction strands (7, 9). More recently, knowledge has been accumulating on the complexity, selectivity, and dynamic character of the intestinal epithelial barrier. Most studies on the paracellular route of transport describe at least two populations of pores regulated by TJs: (1) a high capacity charge selective pore permeable to small ions and small uncharged molecules (referred to as the “pore” pathway), and (2) a much larger low-capacity pore (commonly referred to as the “leak” pathway) permeable to large ions and molecules irrespective of charge. At the molecular level, the first pore is mainly regulated by claudins and the latter by the TJ proteins occludin and the *zonula occludens* (ZO) family.

#### Role of Tight Junctions in Crucial Physiological Processes

The paracellular pathway includes several important aspects: (1) the ability of TJ to restrict paracellular diffusion processes, acting as a paracellular barrier; (2) the ability of the TJ to selectively allow paracellular transport of certain ion species acting as ion channel and (3) the ability of the TJs to maintain cell polarity, by blocking the free diffusion of proteins and lipids between the apical and basolateral domains of the plasma membrane. Moreover, TJ play a crucial role in other relevant physiological processes such as absorption and secretion of water and Ca^2+^ absorption.

#### Absorption of Glucose and Water

When luminal glucose concentrations are low, its absorption involves the transcellular route and the apical Na^+^-glucose co-transporter SGLT1 and the basolateral Na^+^-independent glucose transporter GLUT2 (6). In this environment, absorption of glucose is coupled to an electrochemical gradient; provided by the activity of Na^+^/K^+^-ATPase located at the basolateral membrane (10). Nevertheless, when the luminal concentrations are high as occurs after a meal (concentration range 50–300 mM), about 30% of glucose absorption is mediated via the paracellular route (leak pathway). This pathway includes the phosphorylation of the myosin light chain (MLC) by myosin light chain kinase (MLCK), followed by contraction of the cytoskeleton and the opening of the TJs (11). The osmotic gradient created by transcellular movement of Na^+^ and glucose enhances the paracellular flux of water. This, in combination with the increased paracellular permeability allows paracellular absorption of glucose and other nutrients (12). The Na^+^ absorption is in turn the driving force of the so-called voltage dependent Cl^−^ absorption but it is uncertain whether this route is transcellular or paracellular. It believed that in physiologic conditions when the membrane transporters are saturated, the leak pathway complements the transcellular pathway (active transport) in absorbing water and nutrients (13).

#### Secretion of Water

The entire intestinal epithelium remains hydrated to assure its accurate physiological function. To assure this, there is a constant water secretion in the entire GI tract from the duodenum to the distal colon. The submucosal plexus precisely regulates the secretion of Cl^−^ and consequently Na^+^ and water though by the TJs. The release of vasoactive intestinal peptide (VIP) and acetylcholine (Ach) by submucosal neurons stimulates the enterocyte and Cl^−^ enters by co-transport with sodium and potassium through the basolateral Na^+^-K^+^-2Cl^−^ channel. Then, the Na^+^-K^+^ ATPase pumps Na^+^ back out, and K^+^ is exported via K^+^ channels. The activation of VIP receptors stimulates the adenylyl cyclase to generate cytosolic cAMP that activate the cystic fibrosis transmembrane conductance regulator (CFTR), resulting in secretion of Cl^−^ into the lumen. The activation of muscarinic receptors by Ach induces an increase in intracellular Ca^2+^ concentration that activates calcium-activated chloride channels (CaCC) inducing also Cl^−^ secretion. Accumulation of Cl^−^ in the lumen creates an electric potential that attracts Na^+^ through the TJ into the lumen. NaCl creates an osmotic gradient across the tight junction and water is drawn into the lumen. Some pathogenic bacteria produce toxins (for instance cholera toxin) that permanently activate the adenylate cyclase of the enterocyte. This leads to elevated levels of cAMP, causing the continuous opening of the CFTR channel. As a consequence, there is a massive secretion of water, provoking severe diarrhea (14).

#### Absorption of Calcium

Intestinal Ca^2+^ absorption is a critical physiological process for maintaining Ca^2+^ homeostasis in the entire human body and involves both the transcellular and paracellular pathways. In the proximal small intestine, luminal absorption of low Ca^2+^ concentrations involve mainly the transcellular route (vitamin D-dependent) and for high concentrations, the paracellular passive route (vitamin D-independent) is the predominant source for calcium absorption. In contrast, in the distal small intestine Ca^2+^ absorption mainly involves the paracellular route. Despite the physiological relevance of Ca^2+^ absorption, the mechanisms involved are poorly understood. These mechanisms involve different steps which are currently not well-characterized: (1) entrance of Ca^2+^ into the enterocytes through the Ca^2+^-selective transient receptor potential (TRP) channel TRPV6 and the voltage-gated calcium channel Cav1.3, a member of the L-type calcium channel family; (2) movement of Ca^2+^ to the basolateral membranes by binding to the high Ca^2+^ affinity protein calbindin-D 9 k; (3) basolateral extrusion of Ca^2+^ via plasma membrane Ca^2+^-ATPase PMCA1b and (4) Ca^2+^ extrusion into the blood (15, 16). The pore-forming claudin (CLDN)-2 and barrier forming CLDN-12 seem to be crucial in the Ca^2+^ transport through tight junction proteins (17). Research to elucidate whether other proteins of the pore or the leak pathways are involved in Ca^2+^ absorption is needed.

### The Transcellular Pathway: Transcytosis and Posterior Exocytosis

An increased intestinal transcellular permeability found in different disease conditions has been postulated to be critical for the migration of bacteria and bacterial components such as endotoxins across the gut wall. Based on the molecular weight, isolated LPS can potentially cross the epithelium through the paracellular pathway as has been suggested in some studies (18). Nevertheless, the exact mechanism(s) by which endotoxins (LPS) cross the intestinal barrier remain to be elucidated. In contrast to the general belief, recent research in healthy tissue from animals suggests that LPS is not crossing the epithelium via the paracellular pathway (19). Instead, LPS can potentially cross the enterocyte via the different mechanisms involved in endocytosis: (1) clathrin-mediated endocytosis; (2) lipid-raft caveolae- mediated endocytosis; and (3) via the chylomicrons pathway (only in the small intestine) (19, 20). If these findings can be confirmed in disease conditions, they will strongly challenge the broadly extended concept of “leaky gut” (21).

Contrary to the widely held assumption that whole bacteria can cross the intestinal epithelium via the paracellular pathway, basic research strongly supports that endocytosis is the mechanism used for bacteria to translocate to the lamina propria (22). Whole bacterial translocation through the epithelium occurs not only in the Peyer patches but also in the normal epithelium and involves endocytosis. Nevertheless, the specific pathway(s) of endocytosis that might be involved are not well-characterized. Recent research indicates the role of clathrin-dependent endocytosis in bacterial entry (23). Moreover, Pai et al. (24) recently showed that commensal bacteria endocytosis is also involving MLCK activation and MLC phosphorylation (25), followed by contraction of the actomyosin ring. Then, the perijunctional actomyosin ring contraction produces a change in the configuration of the microvilli called brush border fanning that facilitates the contact of the bacteria with the enterocytes. Remarkable is the fact that this mechanism of bacterial internalization is modulated by tumor necrosis factor-like 1A and interferon (IFN)-γ at low concentrations. IFN-γ at high concentrations is a cytokine that also increases intestinal permeability to large molecules (24).

Despite the increased LPS translocation into the blood described in numerous pathologies, it is not clear whether blood endotoxin levels are the result of LPS from the lumen or whether LPS is released by whole bacteria translocated in the lamina propria by the wall breakdown from effective host defense mechanisms and by autolysis (26).

## Methods to Assess Intestinal Permeability

### *Ex vivo* Assessment of Intestinal Permeability With the Ussing Chambers Technique

In 1951, the Danish scientist Hans Ussing performed for the first time electrophysiology in the frog skin (27). Later, this methodology was adapted and used to study other epithelia of different organs including the GI tract.

#### Transepithelial Electrical Resistance and Paracellular Permeability

Currently, there are different commercially available Ussing chamber equipments that are designed to accommodate multiple epithelial sources such as cell cultures grown in membranes, animal tissue and human tissue from resection specimens or endoscopic biopsies. In such a way, each side of the epithelium is perfectly isolated in each hemi-chamber. Epithelia develop *ex vivo* a potential difference (PD) because they have two differential characteristics compared with other types of tissue: polarity and tightness. They are polarized tissues due to the differential expression of ion channels, pumps and transporters in the apical and the basolateral sides: (a) epithelial sodium channels (ENaC), Ca^2+^ activated chloride channels and the cystic fibrosis transmembrane conductance regulator (CFTR) are present in the apical plasma membrane and (b) the Na^+^-K^+^-ATPase ion pump and the Na^+^-K^+^-2Cl^−^ cotransporter in the basolateral side. Epithelia are tight tissues due exclusively to the presence of TJ that selectively regulate the paracellular flux of ions (see Role of Tight Junctions in Crucial Physiological Processes). The presence of polarity and tightness generate a basal PD across epithelial tissue that is measured in the Ussing chamber using voltage electrodes. Then TEER is measured by (a) applying a constant electric current (open-circuit conditions) in the epithelium every couple of seconds with two current electrodes and (b) measuring then the new generated potential PD. TEER (resistance to the current flow) can then be calculated by using Ohm's law (V = I × R, V: voltage or PD, I: intensity of the current, R: resistance) and expressed as Ω × cm^2^. Low TEER values are indicative of increased permeability to ions. Permeability of the epithelium to ions can be also express in milliSiemens (mS) as the conductance (G) of the epithelium, the opposite of the TEER. Then, an increased epithelial permeability to ions is translated as an increase in conductance. TEER measures the net flux of all ions (cations and anions) across the epithelium and reflects the contribution of the paracellular resistance (R_para_) that reflects the resistance of the TJs, the transcellular resistance (R_trans_) that reflects the resistance to ions of the apical (R_api_) and basolateral (R_bas_) membranes and finally the sub-epithelial resistance (R_sub_). In other words, TEER is the reflection of the resistance that the epithelium is exerting against ions to cross from the luminal to the basolateral side. The use of more complex techniques as the one-path and the two-path impedance spectroscopy is able to measure the two components of the R_epi_, the R_para_ and the R_trans_ (R_epi_ = R_para_ + R_trans_) on top of the R_bas_ (28). Unfortunately, these complex impedance methodologies are only available in a few research groups and total TEER measurements are the most commonly used.

Importantly, TEER does not discriminate between the above-mentioned pore and leak pathways since an increased permeability of both pathways will reduce TEER. The permeability of the leak pathway can be better assessed *ex vivo* by measuring the flux of molecules from the luminal to the basolateral side such as EDTA, mannitol, sucrose, inulin and polyethylene glycols (PEG) or dextran molecules of variable sizes (ranging from 4 up to 20 KDa). For that, labeled molecules with radioactive isotopes or with fluorophores applied at the luminal side are commonly used. Samples from the basolateral side are taken at different time points during ~2 h (29).

The short circuit current (I_sc_) is also often calculated during electrophysiological measurements and refers to the amount of current needed to force the PD to 0 mV. This amount of current is continuously adjusted to clamp the epithelium at 0 mV. Cl^−^ and Na^+^, the most common ions in physiological solutions, mainly generate the PD of the epithelium. I_sc_ is the reflection of the net ionic transport through the epithelium and is expressed as μA/cm^2^. During I_sc_ the voltage is clamped to values different from 0 mV enabling to estimate the TEER.

From the simple equation V = I × R, it is obvious that I_sc_ can also be calculated in open-circuit conditions when R and PD are known. This value is often referred as the equivalent short-circuit current (Ieq).

#### Assessment of the Transcellular Route

The transcellular pathway involving endocytic vesicles and posterior exocytosis can also be assessed in Ussing chambers by adding probes of large molecular weight (MW) that are known to cross the intestinal epithelium using this route. Then, samples from the basolateral side are taken at different time points. Probes most commonly used are horseradish peroxidase (HRP, MW: 44 kDa) and dextran-labeled molecules of a molecular weight between 40 and 80 kDa.

The transcellular route is not only used by large peptides and proteins but also for the uptake of whole commensal bacteria into the lamina propria as part of the BT process. This process can be assessed *ex vivo* by luminal addition of commercially available fluorescently labeled, heat- or chemically killed bacteria such us *Escherichia coli* (K-12 strain) and Staphylococcus aureus (30). Alternatively, live green fluorescent protein-incorporated E. coli or to other bacteria can be used (31).

### *In vivo* Assessment of the Intestinal Permeability

#### Directly: Mainly Assessing the Paracellular Pathway

Virtually all *in vivo* methods to assess paracellular intestinal permeability rely on the urinary excretion of orally ingested probes. Several markers, including different sizes of PEG, ^51^CrEDTA, and especially sugars such as sucrose, sucralose, lactulose, mannitol, and rhamnose, have been used, each with advantages and disadvantages. In general, the probe should be absorbed exclusively via the paracellular pathway, be metabolically inert and freely filtered at the glomerulus without tubular reabsorption (32).

**Figure 2:**
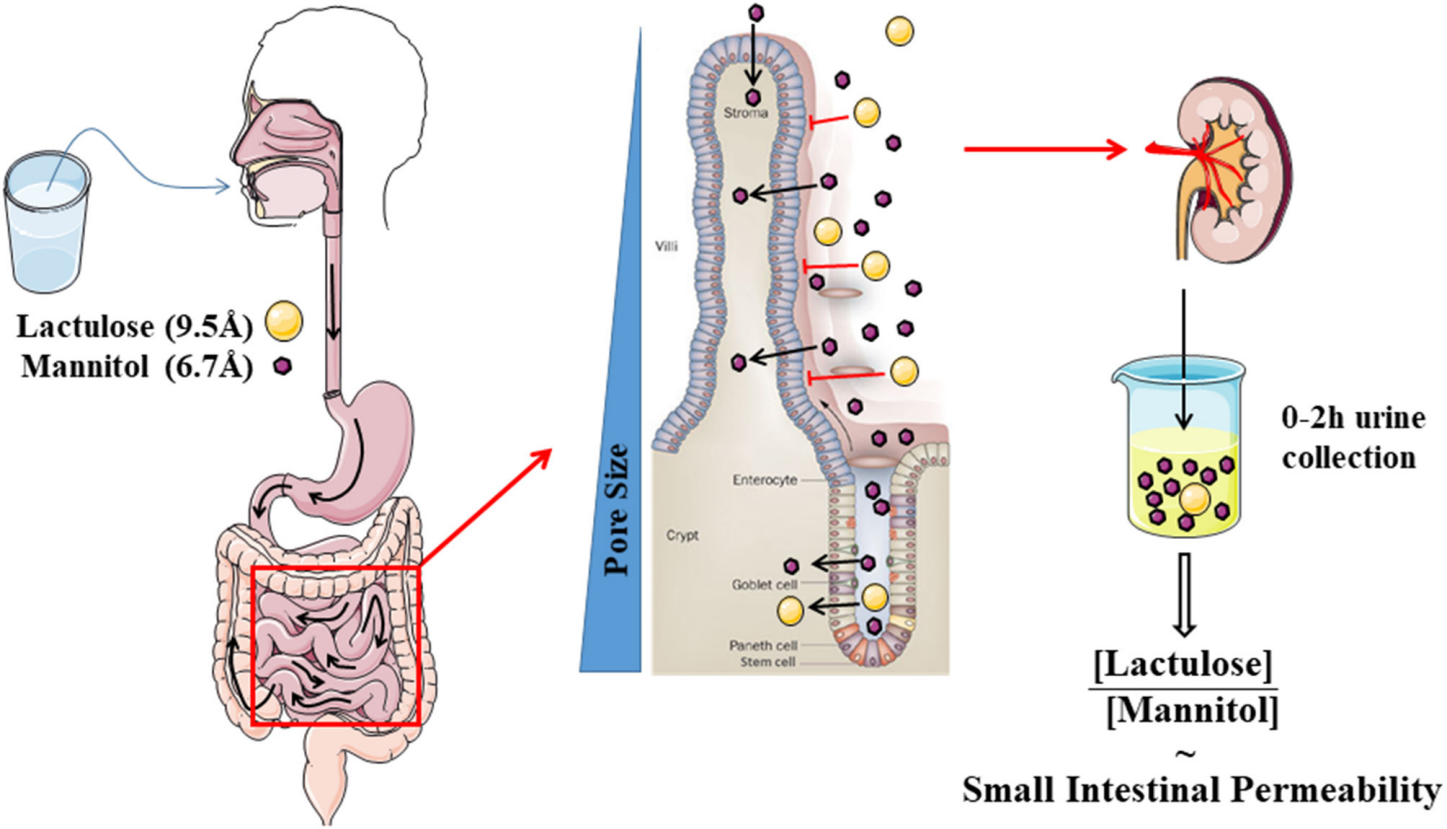
Schematic cartoon of the evaluation of small intestinal permeability by using the lactulose- mannitol test.

The most commonly used test evaluates the differential urinary excretion of the disaccharide lactulose and the sugar alcohol mannitol. Many studies have used the urinary ratio of lactulose to mannitol (LMR) in order to correct for differences in absorption kinetics through gastrointestinal motility and alterations in renal excretion. The LMR in a urine collection of 0–2 h after ingestion is generally accepted as a read-out of small intestinal permeability (13) (Figure 2). A study using radio-isotopes has confirmed that a large proportion of the ingested probe is in the small intestine during this period (33). During 2–8 h, a mixed small intestinal and colonic presence is detected while the 8–24 h collection is most representative for the colonic permeability. However, lactulose and mannitol will be degraded by the colonic microbiota, and hence are not suited to evaluate colonic permeability (34). Other markers including sucralose, PEG, and ^51^CrEDTA are resistant to fermentation and can be used to measure total gastrointestinal permeability with longer urine collections up to 24 h (32). Sucrose is quickly hydrolyzed to fructose and glucose by the digestive enzyme sucrase in the small intestine, which provides the opportunity to estimate gastric and proximal duodenal permeability by measuring urinary sucrose excretion (35). The quantification of urinary sugars is not a standard lab test and should be carefully validated before implementation (36). Especially for lactulose, the urinary recovery can be close to the limit of quantification. Increasing the ingested dose above 5 g should be avoided since this will alter intestinal motility and water handling through its osmotic effect.

The Mayo clinic group has proposed the fractional excretion of mannitol, rather than the LMR to evaluate small intestinal permeability (33, 37, 38). Moreover, ^13^C mannitol has been suggested as an alternative to the common ^12^C mannitol because of the contamination with the latter from food sources in which mannitol is used as a sweetener (e.g., chewing gum) (37–39). However, until now, the test has only been used in healthy volunteers and patients with diarrhea-predominant IBS in whom an increased fractional mannitol excretion but no increased LMR was demonstrated in the 0–2 h collection (33). The effect of treatment on the mannitol excretion has only been evaluated in one study in which gluten induced a higher mannitol excretion and a higher LMR (40). The performance of the mannitol excretion test in other disease conditions and treatments will need to be evaluated and compared against the multitude of studies using a combined assessment of lactulose and mannitol excretion (13).

The truth of the matter is that the permeability pathways of many of the probes are still unclear. The hypothesis behind the LMR is that the smaller mannitol (6.7 Å) can pass via the pore pathway and can be considered as a marker for the total small intestinal surface, while the larger disaccharide lactulose (9.5 Å) will only pass the larger pores of the leak pathway in the crypt region and at sites where the barrier is damaged (13). However, the evidence supporting this hypothesis is scarce, leading to controversy on the validity and especially the interpretation of the lactulose-mannitol test (21). Some authors have suggested that mannitol can also pass transcellularly, although this is not backed up by experimental evidence and is not generally accepted (32). Nevertheless, the urinary recovery of mannitol is 100-fold higher than lactulose, which cannot be sufficiently explained by the size difference (21, 41). Intriguingly, when using the same probes *ex vivo* in Ussing chambers, the difference is much less pronounced with a LMR of ~0.8 *ex vivo* vs. 0.03 *in vivo* (42). The authors hypothesized that this difference is related to an increased absorption of mannitol *in vivo* through solvent drag at the tip of the villi.

Confocal laser endomicroscopy (CLE) is a more recent method to directly assess intestinal barrier function. After intravenous administration of fluorescein, enhancement of the gaps between epithelial cells (1), leakage of fluorescein into the bowel lumen (2), and cell shedding (3) can be directly visualized using a confocal probe equipped with a 488 nm laser (43, 44). Using these three features, increased leakiness has been reported in a variety of conditions, including IBD (45, 46), IBS (47), and acute pancreatitis (48). Moreover, two studies from the same group demonstrated increased duodenal permeability, assessed by CLE, in IBS patients upon perfusion of diluted food antigens (49, 50). However, it is important to keep in mind that CLE visualizes a basolateral (subepithelial) to apical (luminal) flux of a small molecule (fluorescein) of which the correlation to luminal to subepithelial passage of luminal compounds, which is hypothesized to trigger a mucosal immune response and symptoms, is still unclear. Only one study in patients with gastro-esophageal reflux disease (GERD) took the effort of comparing CLE to classical Ussing chamber experiments, which showed no differentiation between GERD and controls by probe-based CLE and no correlation of CLE findings and *ex vivo* permeability (51).

Finally, mucosal impedance testing is another tool to directly evaluate mucosal integrity, although most studies are limited to the esophagus. Impedance is the resistance—or inverse of conductivity—of an alternating electrical current between two adjacent electrodes on a luminal probe. Ten years ago, our group validated this technique to assess esophageal mucosal integrity by showing a correlation to *ex vivo* measurements and a decrease after esophageal perfusion with acidic solutions (52). Since then, it has become clear that baseline impedance, measured by multichannel intraluminal impedance (MII) testing is lower in GERD patients compared to healthy volunteers and improves with medical and surgical therapy (53, 54). More recently, a lower duodenal and jejunal baseline impedance was also demonstrated in patients with functional dyspepsia, corroborating previous *ex vivo* studies (55). The technique has recently been incorporated on a balloon that can be inserted through the biopsy channel of a standard endoscope. By inflating the balloon, the electrodes are brought into contact with the mucosa, and impedance is registered for 90 s as a read-out for mucosal integrity (56, 57). Studies in other segments of the GI tract and comparison of this short measurement to *ex vivo* permeability areawaited.

#### Indirect Assessment of Mucosal Integrity by Potential Blood Biomarkers

Both ex *vivo* and the direct *in vivo* tests for intestinal permeability are time and labor-intensive and are not readily available in most labs. Therefore, there is a growing interest in the validation of blood or urinary biomarkers for intestinal permeability. Strategies include the detection in the blood of molecules normally present in the intestinal lumen as a sign of impaired barrier function (e.g., LPS) or the detection of increased levels of proteins which are components of the intestinal barrier [intestinal fatty-acid binding protein (I-FABP) or tight-junction molecules] signaling damage to the intestinal wall or finally higher blood concentration of barrier-regulating proteins, i.e., zonulin. Unfortunately, with the possible exception of LPS and I-FABP, validation of these assays against standard permeability measurements and proper clinical validation studies in large groups of patients are missing and can therefore not be recommended to replace the direct permeability tests described above.

LPS is a 10–20 kDa protein located in the outer membrane of gram-negative bacteria, which is mainly transported across the epithelium via the transcellular route (58). Increased levels of LPS as a sign of barrier dysfunction have been reported in a variety of conditions, including cirrhosis (4), IBD (5), and IBS-D (6). To circumvent the pitfall of LPS-contamination of common lab equipment, LPS-binding protein (LBP) has been proposed as alternative non-invasive markers indicating increased transepithelial uptake of LPS (59). Even if LBP most likely evaluates the transcellular rather than paracellular pathway, a recent study demonstrated a significant correlation between LBP levels and the LMR in normal weight and obese subjects (60). Similarly, soluble CD14 (sCD14) can also bind LPS in the systemic circulation and is also used as a proxy readout of LPS-activity, although they are not always correlated (61).

I-FABP is a cytosolic protein present in differentiated enterocytes of the small intestine and to a lesser extent in the colon. In normal conditions, this protein is present in low amounts in the circulation, but upon damage to the intestinal barrier, it is released into the bloodstream (62). Increased I-FABP levels have been demonstrated in intestinal ischemia (63, 64), celiac disease (65), necrotizing enterocolitis (66), etc.

A limited number of studies have reported increased systemic levels of claudins as a marker for impaired intestinal barrier function, e.g., increased serum claudin-5 in children with attention deficit hyperactivity disorder (67) and increased urinary claudin-3 in patients with active Crohn's disease which was associated with decreased claudin-3 staining in intestinal biopsies (68). However, validation of these markers against the gold standard, i.e., Ussing chambers, is still lacking.

Finally, zonulin has become a widely used biomarker for intestinal permeability. In 2000, the Fasano lab discovered zonulin as the analog of the prokaryotic zonula-occludens toxin (Zot) in primates (69), and was later identified as prehaptoglobin-2 (70). Zonulin was shown to reversibly open tight-junctions in a protease-activated receptor 2 (PAR2) and epidermal growth factor receptor (EGFR) dependent manner resulting in dislocation of ZO-1 from the tight junction (71). Increased zonulin concentrations have been reported in many conditions, including celiac disease, type 1 diabetes, IBD, obesity, schizophrenia, etc. (72). However, it has become clear that the most widely used enzyme-linked immunosorbent assays (ELISA) do not detect zonulin but complement C3 and possibly properdin, a related molecule from the same family with unclear functional effects (73, 74). A more recent study also confirms that some zonulin ELISA kits are not specific and that increased blood zonulin levels in patients with IBS are not associated with colonic paracellular permeability assessed in Ussing chambers (75). Therefore, the available literature on zonulin as a non-invasive marker for disease characterized by barrier dysfunction should be interpreted with extreme caution.

Table 1 summarizes the different methods described above to evaluate intestinal permeability.

**Table 1:** Summary table of the different methods to assess intestinal permeability.

**Method**	**Principle/description**	**Application**	**Advantages/disadvantages**
•Ussing chambers	- Measures the epithelial permeability to ions by assessing TEER - Measures the epithelial permeability to molecules of different molecular sizes to assess the paracellular and transcellular pathways	Used to assess different aspects of the epithelial integrity *ex vivo* in endoscopic biopsies or resection specimens of different regions of the GI tract	A: gold standard technique for assessing epithelial integrity (intestinal permeability) D: trained researchers and investment in the equipment. D: time and labor-intensive and are not available in most labs
•Differential urinary sugar excretion (most commonly lactulose-mannitol but can include other sugars like sucrose, sucralose, rhamnose)	- Measures the paracellular permeability of the epithelium to different sugars after drinking a sugar cocktail. Sucrose discriminates the paracellular permeability of the gastroduodenal region, lactulose/mannitol the small intestine permeability, and sucralose the colonic/whole GI tract permeability	Used to assess the permeability of the GI tract by measuring in urine the concentrations of the different sugars administered at different fractions of time	A: low cost of the test, a large number of subjects can be included D: determinations of sugar concentrations in urine require investment in a HPLC or LC-MS equipment and trained researchers. D: only measures the paracellular permeability D: time and labor-intensive and not readily available in most labs
•Confocal laser endomicroscopy	- Measures the leakage of fluorescein after intravenous administration visualized using a confocal probe equipped with a 488 nm laser	Used to assess three parameters: the enhancement of the gaps between epithelial cells, leakage of fluorescein into the lumen, and cell shedding	A: relatively easy to perform D: trained researchers and investment in the equipment D: assesses the permeability from the basolateral compartment to the lumen
•Mucosal impedance testing	- Measures mucosal impedance (the equivalent of the resistance for an alternating current) for several seconds through a probe inserted in the biopsy channel of a standard endoscope	Used and validated to assess epithelial integrity in the esophagus. of GERD patients, patients with eosinophilic esophagitis, and other esophageal disorders	A: can be performed during a routine endoscopy D: short assessment of the epithelial integrity (max. 90 s) D: values are the reflection of the epithelial permeability to ions D: validated in the esophagus but not in other parts of the GI tract
•Serum biomarkers: LPS; LBP, sCD14, I-FABP, zonulin	- Determination of serum/plasma concentrations using ELISA	Used to determine blood protein concentrations indicative of bacterial translocation, epithelial damage…	A: reasonable cost and relatively easy to perform in large amounts of samples D: some commercially available ELISAs do not detect the target protein or detect related molecules D: validation of most of these biomarkers against standard permeability measurements and proper clinical validation studies are lacking

*GI, gastrointestinal; A, advantages; D, disadvantages; GERD, gastro-esophageal reflux disease; HPLC, high performance liquid chromatography; LC-MS, liquid chromatography–mass spectrometry; LPS, lipopolysaccharide; LBP, LPS binding protein; I-FABP, intestinal fatty-acid binding protein; sCD14, soluble CD14; ELISA, enzyme-linked immunosorbent assay*.

## Intestinal Permeability in Selected GI Diseases

### Celiac Disease

Celiac disease (CeD) is an immune-mediated enteropathy, triggered by dietary gluten—an alcohol-soluble protein fraction rich in glutamine and proline in wheat, barley, and rye—in genetically predisposed individuals (76). To date, celiac disease is the condition in which the pathogenic role of an impaired barrier function is best established and where steps are being made to translate this finding to a therapeutic target. In the late seventies, Cobden et al. found increased cellobiose excretion and decreased mannitol excretion in a 5 h urine collection in patients with active celiac disease (77). The decreased absorption was attributed to the reduced intestinal absorptive surface of the atrophic mucosa and forms the basis of using mannitol as a marker for intestinal surface area in the LMR. Similar data were presented in a study of 13 untreated CeD patients using a lactulose/rhamnose test (78). Gastroduodenal (sucrose excretion) (79) and whole gut permeability (^51^Cr-EDTA) (80) were also increased in patients with active CeD. These data were confirmed by a lower TEER in Ussing chambers (81, 82) and a decreased number of tight-junction strands shown by freeze-fracture electron microscopy (83). At the molecular level, lower levels of the sealing claudins-3,−5, and−7 and more pore-forming claudin-2 were reported (82). Moreover, in celiac disease claudin-5,−15, ZO-1 and occludin were displaced from their normal position at the tight junction (82, 84).

Gluten ingestion is causally related to the impaired intestinal barrier function in CeD. A single 30 g dose of gluten caused a transient increase in cellobiose excretion in well-controlled patients with normalization within 1 week (85). Moreover, with a gluten-free diet a fast improvement of the cellobiose excretion and a slower recovery of the mannitol excretion—supposedly a marker of regeneration of the intestinal villi—was observed (85). However, in several studies only a partial improvement but not normalization of the barrier was found on a long-term gluten-free diet (79–81), suggesting either that inadvertent dietary mistakes are made or that a barrier defect is a primary event in the pathophysiology of CeD. The latter hypothesis is also suggested by an intermediate (between healthy controls and CeD patients) small intestinal barrier function (LMR) in relatives of CeD patients (86).

The barrier defect in CeD is hypothesized to contribute to diarrhea by a paracellular flux of water and solutes (87). The controversy remains whether the impaired paracellular barrier plays a role in the transepithelial passage of gliadin. Recent data suggest a transcellular passage, dependent on binding to secretory immunoglobulin A (sIgA) and the transferrin receptor CD71 (88).

Two decades ago, increased zonulin protein concentration was shown in patients with active CeD by the group of Fasano (89). Gliadin induced release of zonulin by binding to CXCR3 and elevated permeability *ex vivo* in biopsies of healthy volunteers and patients with quiescent CeD (90, 91). However, both the baseline permeability and permeability after the addition of gliadin were higher and the luminal zonulin release was more pronounced and prolonged in patients with CeD. It is important to notice that these original studies were not performed with the previously mentioned commercial flawed ELISA (73, 74).

Comparison of the amino-terminal end of zonulin and the active fragment of Zot (92) revealed a conserved common motif (69, 93). A synthetic octapeptide (GGVLVQPG), named FZI/0 (92, 94), AT1001 (95, 96), and most recently larazotide (97, 98), corresponding to the amino acids 8–15 of this fragment, did not affect permeability, but pretreatment offered significant protection against the effect of subsequent treatment with purified Zot or zonulin (69, 92). To confirm the contribution of zonulin to early permeability changes in celiac disease, 20 patients on a gluten-free diet were randomized to a 3-day larazotide (12 mg once daily) or placebo treatment with a 2.5 g oral gluten challenge on the second day (96). Intestinal permeability was tested daily by a lactulose/mannitol urinary excretion test. In the placebo group, a significant 70% increase in intestinal permeability was observed after the gluten challenge in contrast to the AT1001 treated patients in whom no changes in permeability were observed. However, the difference between both groups failed to reach statistical significance. Leffler et al. evaluated a 2-week treatment with different doses of larazotide, ranging from 0.5 to 8 mg t.i.d., with daily gluten challenge in 86 celiac disease patients on a gluten-free diet (97). The primary endpoint, a difference in intestinal permeability, was not met in this study with large variability in the LMR. However, significantly fewer gastrointestinal symptoms were observed in patients in the active treatment arm (97). In a second study from the same group, evaluating 1, 4, and 8 mg of larazotide t.i.d. vs. placebo in 184 patients with daily gluten challenge for 6 weeks, gastrointestinal symptoms and anti-tissue transglutaminase IgA antibodies were lower in the active treatment groups. In contrast, and similar to the previous study, the urinary LMR was similar in both groups (98). Finally, 0.5 mg of larazotide t.i.d., but not the higher doses, improved symptoms in CeD patients with persistent symptoms despite following a gluten-free diet (99).

### Inflammatory Bowel Disease

Inflammatory bowel disease (IBD) includes two chronic intestinal inflammatory conditions: Crohn's disease (CD) and ulcerative colitis (UC), characterized by an overactivation and dysregulation of the immune system with a relapsing-remitting pattern. Inflammation in UC is restricted to the superficial layers of the colon whereas, in CD, inflammation is transmural and can affect different regions of the GI tract including the upper part. The exact IBD pathogenesis is unknown, but mounting pieces of evidences indicate a complex interaction between the genome, the exposome, the microbiome, and the immunome (100).

The first attempts to measure intestinal permeability in IBD were performed in the late seventies by rectal instillation of radioiodine-labeled albumin and by measuring later the radioactivity in the plasma in healthy subjects and patients with UC (101). Intestinal permeability using sugar excretion tests (102) and ^51^Cr-EDTA (103) started to be used in the eighties mainly with small groups of patients. From this period, two relevant studies using a larger amount of subjects clearly showed that patients with IBD have an altered intestinal barrier. Total gut permeability using the oral administration of 99mTc-DTPA was increased in patients with CD and UC with both active and inactive disease. Interestingly, the authors found that the degree of intestinal and colonic inflammation was associated with permeability measurements (104). Jenkins et al. showed that the permeability to the oral administration of ^51^Cr-EDTA was increased in patients with small bowel disease and patients with colonic disease. In contrast, only patients with colonic disease had an increased permeability when ^51^Cr-EDTA was administered in the rectum. These findings suggest that the inflamed colon is the site of increased intestinal permeation (105). Since then, a large number of studies have been shown an increased gut permeability in IBD but discrepant findings are reported in different studies. These differences can be explained, at least in part, by the different probes used for the assessment (106). It has been stated and assumed by the majority of the research community that *in vivo* permeability is also increased in first-degree relatives of patients with IBD. Nevertheless, most of the studies do not support this. An increased intestinal PEG-400 permeability in first-degree healthy relatives of Crohn's disease patients was described for the first time in 1986 (107), whereas three other studies could not reproduce these findings by using the same probe (108–110). In addition, no differences were found between healthy relatives and control subjects when the LMR (110–112) or other sugars (110) were used. Interestingly, *in vivo* studies also show that the L/rhamnose ratio was restored with the induction of remission with an elemental diet in patients with active CD (113) and the urinary excretion of ^51^Cr-EDTA was normalized by an anti-TNF-α therapy (114). Moreover, some studies indicate that the increased intestinal permeability assessed in patients in clinical remission predicts a high risk of early relapse (115).

The first study *ex vivo* in Ussing chambers (1999) evaluating the epithelial barrier function in colonic tissue from UC patients with mild or moderate macroscopic disease activity showed a 50% decrease of the total electrical wall resistance (assessed by transmural impedance analysis) when compared to healthy subjects. This reduction was concomitant with increased paracellular permeability of mannitol. Moreover, the strand numbers of the TJs were decreased in UC suggesting a down regulation (116). Interestingly, Söderholm's group in Sweden showed that the TEER and the paracellular passage of ^51^Cr-EDTA and FD4 are not altered in the sigmoid colon of UC patients in remission but they have an increased transepithelial flux of the protein antigen HRP. Nevertheless, the same group recently showed that UC patients in remission have a reduced TEER and an increased paracellular passage of ^51^Cr-EDTA (117). The reason for this discrepancy is not known but could be related to differences in patient cohorts. Electrophysiological and molecular data discussed here contribute to a better understanding of the mechanisms for the increased permeability found *in vivo* and highlight the relevance of the transcellular route in UC.

Studies in CD have been performed in both affected and macroscopically non-inflamed tissues. The total electrical resistance, TEER, in the colon of CD patients with mild or moderate inflammation did not differ between patients with active and inactive disease (remission) when compared to control subjects. In contrast, the epithelial resistance, R_ep_, evaluated by transmural impedance analysis, was reduced in CD patients with active disease only (118). Occludin, claudin-5, and claudin-8 were found to be downregulated and redistributed, whereas claudin-2 (pore-forming TJ protein) was strongly upregulated. In the same direction, Söderholm et al. showed that the TEER of ileal inflamed tissue from CD patients is not altered but the paracellular permeability to ^51^Cr-EDTA is increased (119). The epithelium of the non-inflamed ileum shows a normal permeability to ions and a normal paracellular permeability to ^51^Cr-EDTA as compared to controls (119, 120) but is more susceptible to a challenge with sodium caprate indicating that TJs in the non-inflamed ileum of CD are more reactive to luminal stimuli contributing to the development of mucosal inflammation (120). Moreover, the non-inflamed ileal epithelium has an increased transcellular passage to protein antigens (119, 121) and increased transcellular uptake of non-pathogenic E. coli in the follicle-associated epithelium (FAE) in CD but not in UC (30, 122). Both transcellular mechanisms seem to be mediated by tumor necrosis factor alpha (121, 123).

An elegant study by Pastor-Rojo et al. showed that markers of an impaired barrier function associated with bacterial translocation as LPS and LBP are increased in the serum of CD patients with active and inactive disease whereas they are only increased in UC patients with active disease. Moreover, serum sCD14 levels are only increased in active CD and UC. Serum levels of these markers recovered after treatment to normal levels, although less completely in Crohn's disease (5). Hence, different treatment strategies in IBD patients do not only ameliorate the paracellular permeability (114) but also the transcellular uptake of luminal bacteria and bacterial components (5, 123). A more recent study has shown that the potential intestinal permeability biomarker I-FABP did not differ between endoscopic active disease and remission in both CD and UC (124).

In summary, the inflamed colonic and ileal epithelia from CD patients have a dysregulation of both the pore and the leak pathway. Remarkably, an altered epithelial barrier function has been found in non-inflamed ileal regions in CD. This alteration is characterized by an increased transcellular permeability to protein antigens and whole bacteria translocation in the FAE. The latter may lead to the increased load of commensal bacteria observed in inflamed and non-inflamed mucosa (125) and to the increased concentrations of LPS and LBP found in the blood of CD patients (5).

### Chronic Alcoholic and Metabolic Liver Disease

Metabolic dysfunction-associated liver disease (MALFD), previously known as non-alcoholic liver disease (NAFLD) is the most common cause of chronic liver disease in the Western world with a global prevalence of 25% in the adult population (126). MAFLD covers a spectrum of disease stages ranging from simple steatosis over non-alcoholic steatohepatitis (NASH) to fibrosis and cirrhosis. Chronic and excessive alcohol use causes alcoholic liver disease with a similar progression from steatosis to alcoholic steatohepatitis (ASH) and cirrhosis.

Even if the pathophysiology of both conditions is not fully elucidated and not identical, the role of an impaired intestinal barrier function and altered microbiota in the pathogenesis and progression of chronic liver disease is increasingly recognized as a key player. This close and bidirectional interaction between the liver and the intestinal tract is termed the “gut-liver axis” (127–129). The central hypothesis of the gut-liver axis is that translocation of luminal microbiota or microbial products (pathogen-associated molecular patterns or PAMPs) through an impaired intestinal barrier function is an early step in the pathogenesis of chronic liver disease (4). A hepatic immune response will be triggered by the interaction of PAMPs (e.g., LPS and bacterial RNA) with pathogen recognition receptors such as Toll-like receptor (TLR)4 on hepatic Kuppfer cells, the resident macrophages in the liver sinusoids (130). This immune response will then lead to progressive liver injury and ultimately to fibrosis by activation of hepatic stellate cells (131–133).

Several groups have investigated *in vivo* intestinal permeability in MAFLD: small intestinal permeability by lactulose/mannitol (134–140) or lactulose/rhamnose (141) and whole gut permeability by ^51^Cr-EDTA (142) or sucralose (140). A recent systematic review and meta-analysis demonstrated increased small intestinal and whole gut permeability in MAFLD patients, even if this was not confirmed in all individual studies (134, 140, 141). All described studies evaluating small intestinal permeability used a 5 or 6 h urinary collection to assess small intestinal permeability, which may have confounded the results since a large amount of the sugars were probably already localized in the colon by the end of the urine collection (33). Increased zonulin levels were also reported in five pediatric and adult MAFLD studies, but because of the flawed commercial zonulin ELISA, these reports do not advance the field (73, 74). Taking into account these limitations, one of the more informative studies was performed by Miele et al. who showed increased ^51^Cr-EDTA in a 24 h urine collection in biopsy proven MAFLD vs. healthy controls (142). Moreover, higher intestinal permeability was present in patients with moderate or severe steatosis vs. patients with mild steatosis but was not associated to hepatic inflammation (NASH). Interestingly, patients with increased intestinal permeability were also more likely to have a pathological glucose breath test, which is indicative of small intestinal bacterial overgrowth (SIBO) (88.8 vs. 29.4% in MAFLD patients with increased vs. normal permeability; *P* < 0.001). Conversely, patients with SIBO also had a higher intestinal permeability. The authors also reported a correlation between impaired permeability and lower nuclear ZO-1 staining, the importance of which is difficult to interpret because its normal localization should be at the level of the tight-junctions (142). In a recent study in liver cirrhosis of any cause, not only urinary ^51^Cr-EDTA excretion was elevated in about half the patients and associated with Child-Pugh state, it was also present in all ascites samples from patients with spontaneous bacterial peritonitis (SBP) (143). Nevertheless, even if 85% of patients with cirrhosis in another study had a pathological LMR, the test failed to predict the development of SBP or survival (144).

In 1984, Bjarnason et al. were the first to describe increased whole gut permeability in patients with alcohol abuse without significant liver disease, measured by a 24 h urinary excretion of ^51^Cr-EDTA, which was confirmed by increased uptake of macromolecules (inulin, EDTA and cyanocobalamin) *ex vivo* in jejunal biopsies (145). Since then, increased whole gut permeability to larger molecular weight PEG molecules (MW 1,500 and 4,000) (146) and increased urinary excretion of sucrose in a 5–12 h collection (147) was reported in chronic alcoholic liver disease patients. Chronic alcohol use can by itself damage the intestinal barrier and increases plasma LPS levels, both of which normalized after a longer period of abstinence (145, 148). However, in a recent study in 106 patients with alcohol use disorder with variable degrees of liver disease, intestinal permeability normalized after a 3-week detoxification program, while markers of bacterial translocation (LBP) remained elevated (149). Moreover, baseline LBP and sCD14 levels were similar in patients with normal (2/3 of patients) and increased intestinal permeability (urinary ^51^Cr-EDTA excretion), highlighting again that bacterial translocation is not a paracellular phenomenon and that one should be careful of connecting clinical outcomes to paracellular permeability tests (149).

Very few studies have directly evaluated intestinal permeability in Ussing chambers. Du Plessis et al. described increased passage of labeled 4 kDa dextrans and a lowered TEER in duodenal biopsies in patients with compensated and decompensated cirrhosis from different etiologies (4). At the molecular level, an increased protein level of claudin-2, a pore-forming claudin, was demonstrated in comparison to healthy controls, although these pores are of insufficient size to allow diffusion of bacterial products such as LPS in decompensated cirrhosis (4). Decreased expression of occludin and claudin-1 in the duodenal mucosa have also been reported in cirrhosis and their expression levels correlated with LPS concentrations, severity of liver disease and portal hypertension (esophageal varices) (150). In a very recent study Haderer et al. reported a decreased mucus thickness in the colon of cirrhotic patients and a breakdown of E-cadherin and occludin by endogenous and newly identified bacterial proteases respectively, which could be future therapeutic targets (151).

Several etiological factors are most likely involved in the pathogenesis of the impaired barrier function in chronic liver disease, but the four most important ones are direct toxicity of alcohol, portal hypertension, alterations in the intestinal microbiome and altered bile acid signaling.

Alcohol and its metabolite acetaldehyde exert a direct toxic effect to the intestinal tight junctions with a redistribution of ZO-1 and occludin (152, 153). These data were also confirmed *in vivo* by duodenal perfusion of 20 g ethanol vs. placebo in healthy volunteers (154). Moreover, increased gene expression of MLCK pointed toward an activation of the leak pathway through contraction of the cytoskeleton. These effects were dependent on the activation of the mitogen activated protein kinase (MAPK) pathway (154). However, since small intestinal permeability was significantly higher in patients with alcoholic liver diseases than in patients with chronic alcohol abuse in the absence of liver disease, the direct barrier toxicity of alcohol does not fully account for the permeability defect (155).

Congestion of the intestinal microcirculation in portal hypertension may cause intestinal and vascular barrier dysfunction. A causal role for portal hypertension in the pathogenesis or further progression of the barrier defect in chronic liver disease is suggested by an improvement of intestinal permeability and LBP levels with non-selective beta-blockers and transjugular intrahepatic portosystemic shunting (TIPS) (156, 157). However, macrophage activation status (sCD163 levels) remained elevated even after normalization of the portal-venous pressure gradient with TIPS (157).

Several studies have shown microbial alterations in MALFD, alcoholic liver disease and acute-on-chronic liver failure which also evolve with disease severity (158–162). However, most studies are based on the analysis of stool microbiota which may not be fully representative of the small intestinal microbiota which can translocate through the intestinal barrier. Recently, Raj et al. investigated the mucosa-associated microbiota in duodenal biopsies of 35 patients with chronic liver disease of different etiologies. Patients displayed a lower microbial diversity and a significantly different composition of the duodenal microbiome with a lower abundance of the genera Moryella, Porphyromonas and Veillonella (163). The lower alpha diversity—but not serum LPS levels—also correlated with increased intestinal permeability, measured by a plasma lactulose/rhamnose ratio 90 min after ingestion of the sugar solution, which is a less validated way to assess intestinal permeability because this one sampling point does not take into account renal clearance. A similar correlation between intestinal barrier function and fecal microbial alterations has also been shown in alcohol-dependent patients (149, 164). How dysbiosis affects intestinal barrier function is largely unknown, but one mechanism is through decreased production of short-chain fatty acids (SCFA) such as butyrate, although this is largely based on preclinical data and would be mainly relevant for colonic barrier function (165).

Finally, the altered microbiome can change the bile acid pool by the conversion of primary to secondary bile acids. The altered bile acid pool is associated with impaired intestinal barrier function (166). Conversely, bile acids also affect the gut microbiota through activation of the innate immune system and production of antimicrobial peptides (127). Finally, decreased activation of the farnesoid X receptor (FXR) in the distal small intestine by bile acids may also contribute to impaired integrity of the intestinal barrier (127).

At this moment, treatments targeting the intestinal barrier in chronic liver disease are still lacking. Preclinical data demonstrate a restoration of intestinal barrier function upon stimulation with FXR agonists, e.g., obeticholic acid (128). However, to the best of our knowledge, clinical data of FXR agonists on barrier function in human chronic liver disease are lacking. In a recent study in 21 patients with MAFLD, fecal microbiota transfer (FMT), administered in the duodenum via endoscopy, improved the LMR in contrast to those who received an autologous FMT. However, there was no effect on insulin resistance or steatosis (167).

### Bile Acid Malabsorption

Bile acids are synthesized in the liver and released into the duodenum to facilitate, through their detergent properties, the solubilization and absorption of fatty acid and monoglycerides in the small intestine. Under normal circumstances, more than 90% of bile acids are reabsorbed in the small intestine and transported back to the liver, a process referred to as enterohepatic circulation. The bile acid pool, estimated at 2–4 grams, circulates six to 10 dimes daily through the enterohepatic circulation. The reuptake of bile acids in the ileum occurs through active transport, for conjugated bile acids, or by passive absorption, for unconjugated bile acids.

Active transport of bile acids from the lumen into the enterocyte occurs through an apical sodium-dependent bile salt transporter in the brush border. Inside the enterocyte, ileal bile acid binding protein allows transfer through the cell and delivery at the basolateral side through organic solute transporters α and β. From there, the bile acids reach the portal circulation and the liver where they are recycled. Passage of bile acids through the enterocyte activates FXR, which promotes the synthesis of fibroblast growth factor 19 (FGF19), which also circulates through the portal vein to the liver where it activates fibroblast growth factor-receptor 4 resulting in inhibition of bile acid synthesis. Binding of FGF to FGFR4 is facilitated by the transmembrane protein Klotho β, and genetic variants in this protein have been associated with colonic transit in IBS with diarrhea. In case of low bile acid absorption, FXR is not activated, FGF19 stays low and bile acid synthesis is stimulated. Bile acids are synthesized from cholesterol and serum levels of 7α-hydroxy-4-cholesten-3-one (C4), an intermediate product of this process, reflect the activity of bile acid synthesis (168).

In case of organic disease, that affects the terminal ileum (e.g., Crohn's disease, ileal resection, etc.), bile acids are not absorbed and enter the colon, where they induce diarrhea. This is referred to as bile acid diarrhea type 1. In case of mutations affecting the function of the bile salt transporter, but especially when the FGF19-driven negative feedback pathway is defective, this leads to bile acid diarrhea type 2 (168). Patients with this type of bile acid diarrhea have significantly lower FGF19 and higher 7a-hydroxy-4-cholesten-3-one (C4) levels than healthy controls (169), which supports the hypothesis that excess synthesis of bile acids may overflow the reabsorption capacity in the ileum and hence allow bile acids to enter the colon in larger quantities.

Bile acids in the colon may facilitate the occurrence diarrhea in a number of ways. Through activation of the G protein-coupled receptor TGR5, expressed on enterocytes, entero-endocrine cells and on enteric neurons, bile acids may stimulate colonic motility (170). In man, colonic or rectal instillation of bile acid solution stimulates propulsive motility (171, 172). Administration of chenodeoxycholic acid accelerated colonic transit in healthy controls (173). Bile acids also promote colonic water secretion, through TGR5-mediated activation of adenylate cyclase, which stimulates chloride secretion through the cystic fibrosis transmembrane conductance regulator (CFTR) (174). In addition, bile acids also inhibit apical chloride/bicarbonate exchange in enterocytes (175).

Several *ex vivo* and *in vivo* studies have demonstrated that bile acids may affect mucosal integrity, in the esophagus and in the small intestine. TGR5-deficient mice have increased colonic mucosal permeability, through disruption of epithelial tight junctions (176). Hence, it has been suggested that bile acid diarrhea also involves increased colonic mucosal permeability. In the rabbit colon *ex vivo*, mucosal permeability was increased by deoxycholic and chenodeoxycholic acid (177). In a mixed cohort of controls and IBS patients, total fecal bile acid content was significantly correlated with mucosal permeability (178).

In IBS-D patients with elevated serum C4 a borderline increased colonic permeability was found based on urinary mannitol excretion (178). However, C4 levels in IBS-D are not significantly correlated to mucosal permeability as assessed by urinary excretion of lactulose/mannitol, and in 23 patients with IBS-D, treatment with colesevelam tended to improve stool consistency but did not alter mucosal permeability as assessed by lactulose/mannitol excretion (173). In a controlled colesevelam trial in 30 patients with IBS-D, colesevelam did not alter mucosal permeability (179). Taken together, these studies do not confirm increased mucosal permeability as a key mechanism involved in bile acid diarrhea.

## Conclusion and Knowledge Gaps

The intestinal barrier function is a complex integrated system composed of several pathways. However, evaluation of the intestinal barrier in clinical studies is usually limited to the assessment of the paracellular permeability. Moreover, not all methodologies utilized in these studies are properly validated against the gold standard, i.e., the Ussing chamber, and therefore caution is needed in the interpretation of these permeability data. In the clinical conditions presented in the review, increased intestinal permeability has been demonstrated, but the central question of whether the barrier dysfunction is a primary event in the pathophysiology or a consequence of the disease is still not resolved in most diseases. Until therapies specifically correcting barrier function, which could demonstrate a causal role for intestinal permeability, become available, the controversy will remain and diagnosis of “leaky gut” has no role in diagnosis or treatment of human disease.

## Author Contributions

TV, JT, and RF: contributed on writing and review the original draft. RF: editing. All authors contributed to the article and approved the submitted version.

## Conflict of Interest

The authors declare that the research was conducted in the absence of any commercial or financial relationships that could be construed as a potential conflict of interest.

## Publisher's Note

All claims expressed in this article are solely those of the authors and do not necessarily represent those of their affiliated organizations, or those of the publisher, the editors and the reviewers. Any product that may be evaluated in this article, or claim that may be made by its manufacturer, is not guaranteed or endorsed by the publisher.
